# Studies on alpha-synuclein and islet amyloid polypeptide interaction

**DOI:** 10.3389/fmolb.2023.1080112

**Published:** 2023-01-30

**Authors:** Ye Wang, Joakim Bergström, Martin Ingelsson, Gunilla T. Westermark

**Affiliations:** ^1^ Departments of Medical Cell Biology, Uppsala University, Uppsala, Sweden; ^2^ Departments ofPublic Health and Caring Sciences, Uppsala University, Uppsala, Sweden; ^3^ Krembil Brain Institute, University Health Network, Toronto, ON, Canada; ^4^ Department of Medicine and Tanz Centre for Research in Neurodegenerative Diseases, University of Toronto, Toronto, ON, Canada

**Keywords:** alpha-synuclein, islet amyloid polypeptide, amyloid, BiFC, cross-seeding

## Abstract

**Introduction:** Parkinson’s disease and type 2 diabetes have both elements of local amyloid depositions in their pathogenesis. In Parkinson’s disease, alpha-synuclein (aSyn) forms insoluble Lewy bodies and Lewy neurites in brain neurons, and in type 2 diabetes, islet amyloid polypeptide (IAPP) comprises the amyloid in the islets of Langerhans. In this study, we assessed the interaction between aSyn and IAPP in human pancreatic tissues, both *ex vivo* and *in vitro*.

**Material and Methods:** The antibody-based detection techniques, proximity ligation assay (PLA), and immuno-TEM were used for co-localization studies. Bifluorescence complementation (BiFC) was used for interaction studies between IAPP and aSyn in HEK 293 cells. The Thioflavin T assay was used for studies of cross-seeding between IAPP and aSyn. ASyn was downregulated with siRNA, and insulin secretion was monitored using TIRF microscopy.

**Results:** We demonstrate intracellular co-localization of aSyn with IAPP, while aSyn is absent in the extracellular amyloid deposits. ASyn reactivity is present in the secretory granules of β-cells and some α-cells in human islets. The BiFC-expression of aSyn/aSyn and IAPP/IAPP in HEK293 cells resulted in 29.3% and 19.7% fluorescent cells, respectively, while aSyn/IAPP co-expression resulted in ∼10% fluorescent cells. Preformed aSyn fibrils seeded IAPP fibril formation *in vitro*, but adding preformed IAPP seeds to aSyn did not change aSyn fibrillation. In addition, mixing monomeric aSyn with monomeric IAPP did not affect IAPP fibril formation. Finally, the knockdown of endogenous aSyn did not affect β cell function or viability, nor did overexpression of aSyn affect β cell viability.

**Discussion:** Despite the proximity of aSyn and IAPP in β-cells and the detected capacity of preformed aSyn fibrils to seed IAPP *in vitro*, it is still an open question if an interaction between the two molecules is of pathogenic significance for type 2 diabetes.

## 1 Introduction

Amyloidoses constitute the largest group among protein-misfolding diseases. More than 36 different amyloid-forming proteins have been identified in humans, each of which links to the development of at least one specific disease (Buxbaum et al., 2022). Alzheimer’s disease (AD), Parkinson’s disease (PD), and Type 2 diabetes (T2D) are three well-known conditions with localized amyloid deposits as a shared pathological mechanism essential for the development of respective diseases. Despite significant differences in the primary structure of amyloid-forming proteins, all amyloid deposits exhibit morphological similarity with unbranching fibrils 8–10 nm in width when examined in the electron microscope, and all have a high affinity for the amyloid dye Congo red.

Parkinson’s disease is the second most common neurodegenerative disorder, which occurs in 0.5%–1% of the population over 60 (Tanner and Goldman, 1996; Tysnes and Storstein, 2017). A progressive loss of dopaminergic neurons in *substantia nigra* (SN) results in the three principal symptoms: resting tremor, bradykinesia, and rigidity (Parkinson, 2002). The formation of intracellular Lewy bodies (LBs) and Lewy neurites (LNs), which mainly consist of fibrillar alpha-synuclein (aSyn), is the pathological hallmark of PD (Spillantini et al., 1997). Mutation in the aSyn gene SNCA can cause familial PD (Polymeropoulos et al., 1997), while the multiplication of SNCA affects the progression of PD in a dose-dependent manner (Singleton et al., 2003; Chartier-Harlin et al., 2004).

T2D is a severe chronic metabolic disease affecting more than 8% of the adult population worldwide (World Health Organisation Diabetes (Internet), 2021). There is no cure for the disease, and many affected will develop long-term health complications. In more than 90% of the patients with T2D, amyloid deposits are present in the islets of Langerhans (Westermark, 1972; Clark et al., 1996), and islet amyloid polypeptide (IAPP) is the main amyloid component. IAPP is synthesized as proIAPP, which undergoes post-translational processing and modifications to gain biological activity (Marzban et al., 2004; Marzban et al., 2005). Both proIAPP and IAPP occur in amyloid deposits in β cells (Paulsson and Westermark, 2005; Paulsson et al., 2006).

Epidemiological studies suggest that diabetes predisposes to neurodegenerative diseases (Ott et al., 1999; Arvanitakis et al., 2004). Previously, we and others have shown that IAPP is present in Aβ deposits in the human brain (Jackson et al., 2013; Oskarsson et al., 2015), and proximity ligation assay (PLA) was used to confirm co-localization of Aβ and IAPP in amyloid deposits in both cortex and blood vessel walls of AD patients (Oskarsson et al., 2015). Recently, we used bimolecular fluorescence complementation (BiFC) and visualized the interaction between IAPP and Aβ in living cells, providing a molecular link between AD and T2D (Wang and Westermark, 2021). Patients with T2D have an increased risk of developing PD (Sandyk, 1993; Hu et al., 2007), and when PD develops, the disease has a more aggressive phenotype (Pagano et al., 2018).

There are studies on molecular interactions between IAPP and aSyn with contradictory results on heterologous interactions and seeding (Horvath and Wittung-Stafshede, 2016; Mucibabic et al., 2020). With this work, we aimed to further investigate the possible interaction between aSyn and IAPP in the formation of amyloid fibrils and investigate if aSyn has a principal function in insulin release. We show that aSyn, in part, occurs in secretory granules of β cells, binds to IAPP, and co-localizes with IAPP intracellularly, but not in the amyloid deposits. *In vitro* studies, IAPP fibrillation is not affected by the addition of monomeric aSyn, while aSyn seeds trigger the fibrillation of monomeric IAPP. Despite the observed physical proximity of IAPP and aSyn, the knockdown of aSyn does not affect β cell function or viability.

## 2 Results

### 2.1 Presence of aSyn in human islets and EndoC-βH1 cells

**FIGURE 1:**
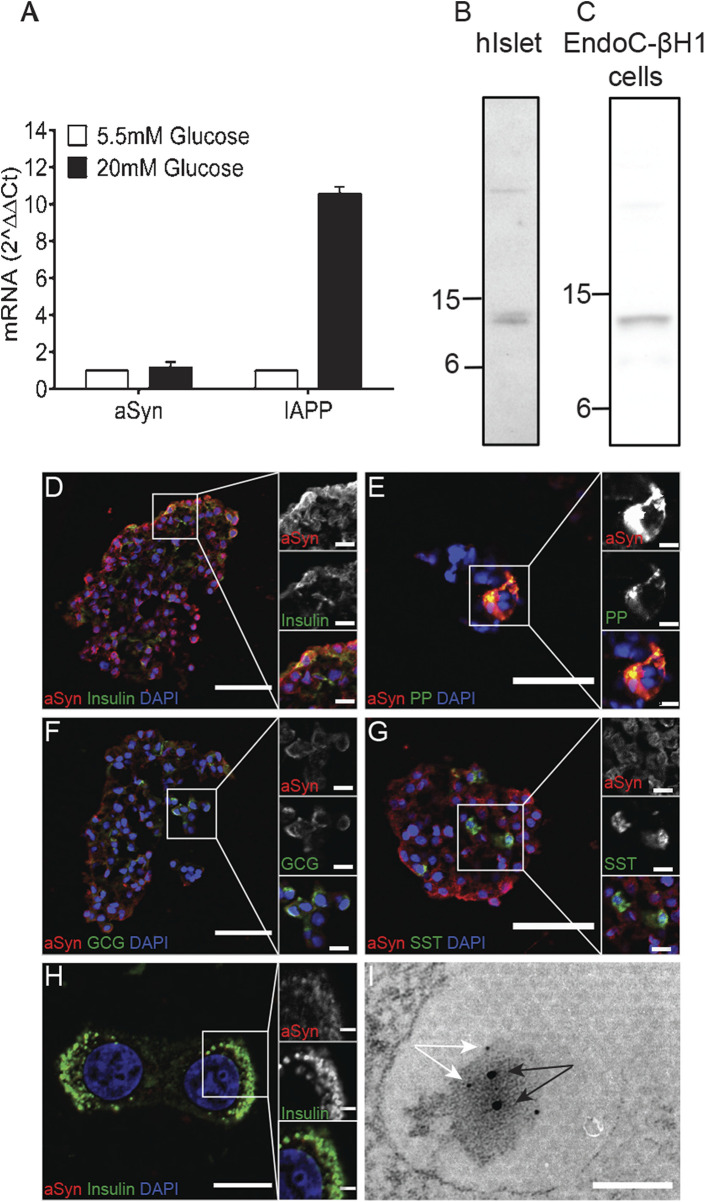
ASyn is present in human islets and human EndoC-βH1 cells. Human islets (*n* = 2) were divided into two groups and cultured in normal glucose (5.5 mM) or high glucose (20 mM) for 2 days before being analyzed for aSyn mRNA and IAPP mRNA. Data were presented as fold changes, and expression of aSyn mRNA and IAPP mRNA were normalized to GUSb and relative to normal glucose. **(A)** Culturing in high glucose slightly increased aSyn mRNA levels by 1.18-fold, whereas the same treatment increased IAPP mRNA by 10-fold. Western blot data confirm the presence of aSyn in **(B)** human islets and **(C)** human EndoC-βH1 cells as 14 kDa band using aSyn-specific antibody Syn-1. The localization of aSyn in human islets was investigated using double-immunofluorescence staining. ASyn reactivity co-localized with **(D)** insulin and **(E)** pancreatic polypeptide, partially co-localized with **(F)** glucagon, but no co-localization occurred with **(G)** somatostatin. The co-localization of aSyn and insulin reactivity was also detected in **(H)** EndoC-βH1 cells. **(I)** In the electron micrograph, aSyn (10 nm gold particles, black arrows) and insulin (5 nm gold particles, white arrows) reactivity were both detected in secretory granules of β cells, especially around the dense core of granules. Scale bars are 50 μm and 10 µm in insets **(D–G)**, 10 μm and 2 µm in inset **(H)**, and 100 nm in **(I)**.

The aSyn gene is ubiquitously expressed with strong enrichment in the cerebral cortex. Analysis of aSyn expression in human islets with quantitative PCR (qPCR) revealed low mRNA expression. In human islets, IAPP expression is restricted to the β cells, and the expression is known to be affected by glucose. The culture of human islets in 20 mM glucose for 48 h resulted in a 10-fold increase in IAPP mRNA concentration compared to islets cultured in 5.5 mM glucose. However, a corresponding rise in aSyn mRNA in response to glucose was not observed, as the aSyn mRNA concentration was only increased 1.18-fold after culture in 20 mM glucose for 48 h (Figure 1A).

aSyn reactivity was detected in the lysate of human islets with the Syn-1 antibody as a 14 kDa band (Figure 1B; Table 1), consistent with the predicted molecular weight. Furthermore, a similar reactivity of aSyn was detected in the lysate of human β cell line EndoC-βH1 (Figure 1C), indicating that at least a portion of the aSyn reactivity from human islets originated from β cells. As determined by ELISA, the concentration of aSyn and IAPP per 10^6 cells were 28.9 μg and 17.6 μg, respectively.

**TABLE 1:** Information on antibodies used for immunofluorescence, proximity ligation assay, Western blot and immunoEM.

Antibodies	Species	If	PLA	WB	ImmunoEM	Supplier
Primary Abs						
Syn-1	mouse	1:50	1:500	1:1000	1:200	BD Biosciences
1338	mouse	1:50	—	—	1:200	R&D System
01B	rabbit	1:50	1:500	—	—	Authors
Glucagon	rabbit	1:1000	—	—	1:200	DAKO
Insulin	guinea pig	1:250	—	—	1:200	DAKO
Somatostatin	rabbit	1:1000	—	—	—	DAKO
Pancreatic peptide	sheep	1:250	—	—	—	DAKO
A110	rabbit	—	1:1000	—	—	Authors
SM1341	mouse	—	1:500	—	—	Acris
Secondary Abs						
goat-anti-rabbit Alexa 488		1:1000				ThermoFisher
goat-anti-guinea pig Alexa 488		1:1000				ThermoFisher
donkey-anti-sheep Alexa 488		1:1000				ThermoFisher
goat-anti-mouse Alexa 555		1:1000				ThermoFisher
goat-anti-rabbit Alexa 546		1:1000				ThermoFisher
anti-mouse HRP				1:1000		DAKO
anti-rabbit 5 nm gold					1:200	BioCell
anti-guinea pig 5 nm gold					1:200	BioCell
anti-mouse 10 nm gold					1:200	BioCell

IF: immunofluorescence, PLA: proximity ligation assay, WB: Western blot.

The aSyn distribution in the islet of Langerhans was showed using double immunofluorescence, where an aSyn-1 antibody was combined with antibodies targeting insulin, glucagon, somatostatin, or pancreatic polypeptide (PP). Reactivity of aSyn co-localized with insulin in most of the β cells (Figure 1D) and with PP in PP cells (Figure 1E). A co-localization with glucagon was observed in a fraction of α cells (Figure 1F), whereas no aSyn reactivity occurred in δ-cells positive for somatostatin (Figure 1G). Double immunofluorescence staining using antibodies targeting insulin and aSyn on EndoC-βH1 cells confirmed the co-localization of insulin and aSyn (Figure 1H).

Identification of aSyn expression in insulin and some glucagon-producing cells prompted us to investigate the subcellular localization of aSyn. With immunoelectron microscopy, co-localization between aSyn and insulin occurred in the dense core region of the secretory granules in β cells (Figure 1I). Similarly, co-localization between aSyn and glucagon appeared in secretory granules of some α cells (data not shown).

### 2.2 aSyn and IAPP interact intracellularly, but aSyn was absent in islet amyloid

**FIGURE 2:**
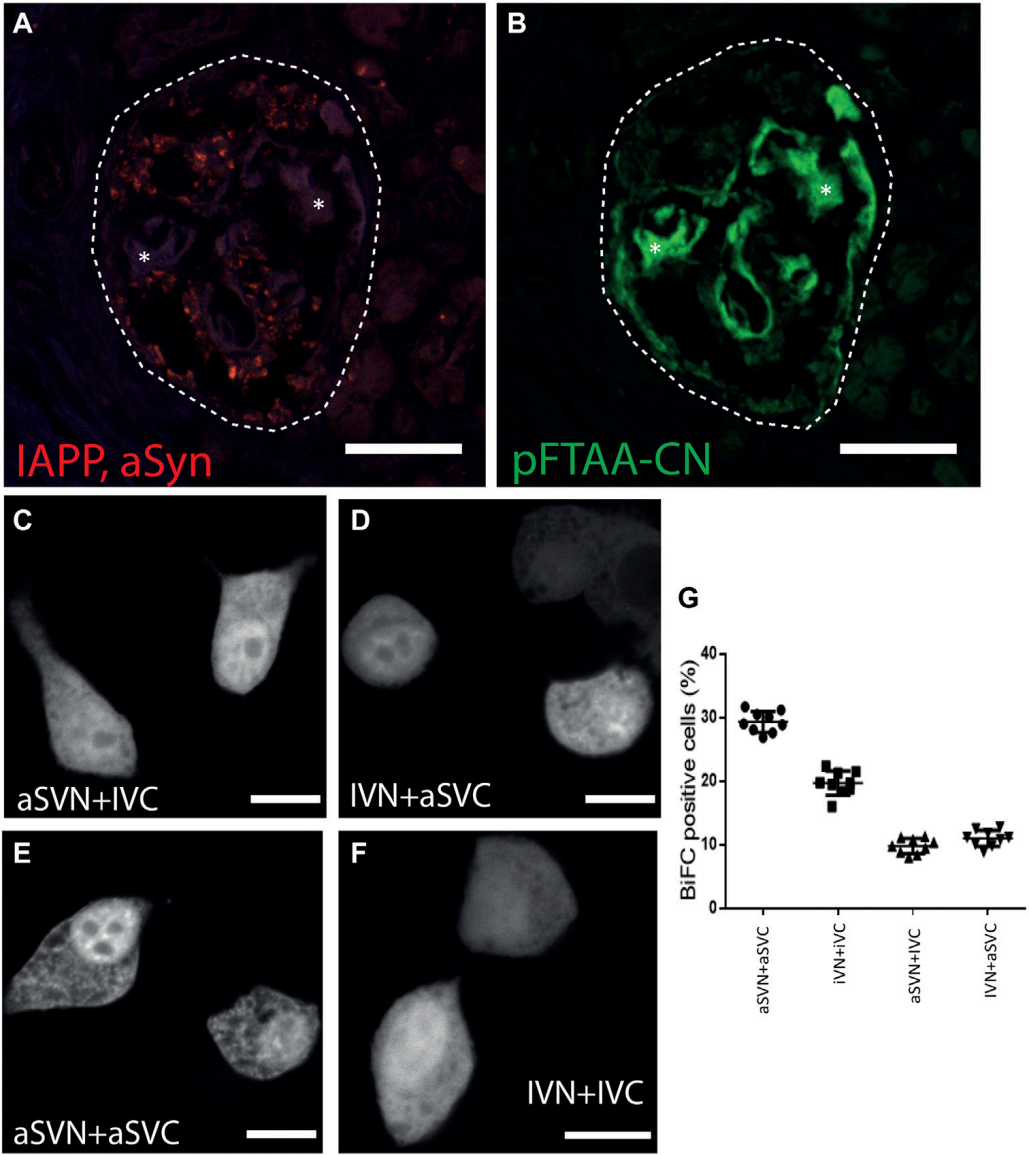
ASyn co-localize with IAPP intracellular but not with islet amyloid. PLA was performed on formalin-fixed paraffin-embedded human pancreas sections using aSyn and IAPP antibodies. **(A)** Positive PLA signals (red dots) were detected in human islets, indicating that aSyn and IAPP were located within 40 nm. After PLA, the same sections were incubated with **(B)** pFTAA-CN to visualize islet amyloid, which demonstrated that PLA signals were not present in the islet amyloid area. BiFC constructs were expressed in HEK 293 cells to study aSyn (61–95) and IAPP interaction. Confocal images of BiFC-positive cells expressing aSVN + IVC in **(C)**, and IVN + aSVC in **(D)**, aSVN + aSVC in **(E)** and IVN + IVC in **(F)**. In **(G)**, percentages of BiFC-positive HEK293 cells were determined by flow cytometry. Asterisks denote corresponding areas in both pictures. Scale bars are 50 µm in **(A, B)**, 10 µm in **(C–F)**.

Proximity ligation assay (PLA), an antibody-based detection system, was used for co-localization studies of IAPP and aSyn in pancreas sections from patients with T2D. After combining IAPP and aSyn antibodies, a positive PLA signal seen as red dots appeared in pancreatic islets (Figure 2A; Table 1), while no reactivity occurred in the exocrine pancreatic tissue. We investigated if aSyn deposited together with IAPP at intra- and extracellular locations by staining the PLA sections with the amyloid ligand pFTAA-CN, a luminescent conjugated oligothiophene dye (Nilsson et al., 2018). The results showed that the PLA signals were restricted to the islet cell area but absent in islet amyloid deposits (Figure 2B). The localization of the PLA signal suggests that aSyn and IAPP occur within 40 nm intracellularly, but aSyn is not present in the extracellular amyloid deposit.

The BiFC technique was applied to investigate if IAPP and aSyn interact on a molecular level. The hydrophobic non-amyloid-B component (NAC) region of aSyn, corresponding to residues 61–95 of aSyn, has been shown to form the core of aSyn fibrils (Han et al., 1995; Iwai et al., 1995). This 35-amino acid long fragment is more comparable in size to the 37-amino acid long IAPP than full-length aSyn and was therefore used in our study. The code used to distinguish BiFC constructs were designated as follows: aS for aSyn (61–95), I for IAPP, V for Venus, N or C for N-terminal (1–173) or C-terminal (155–239) fragments of Venus, respectively. A diffuse cytoplasmic fluorescent signal appeared in HEK293 cells transfected with aSVN + IVC (Figure 2C) and IVN + aSVC (Figure 2D), supporting interaction between aSyn (61–95) and IAPP, which suggested that aSyn (61–95) and IAPP could interact in a parallel orientation. As expected, the expression of the homologous combinations aSVN + aSVC (Figure 2E) and IVN + IVC (Figure 2F) resulted in fluorescent signals. Flow cytometry analysis revealed a fluorescent signal in 29.3% of cells transfected with aSVN + aSVC, and 19.7% of cells transfected with IVN + IVC. In contrast, transfection with aSVN + IVC and IVN + aSVC resulted in fluorescent signals in 9.8% and 11% of cells, respectively.

### 2.3 Monomeric aSyn does not promote IAPP fibril formation *in vitro*

**FIGURE 3:**
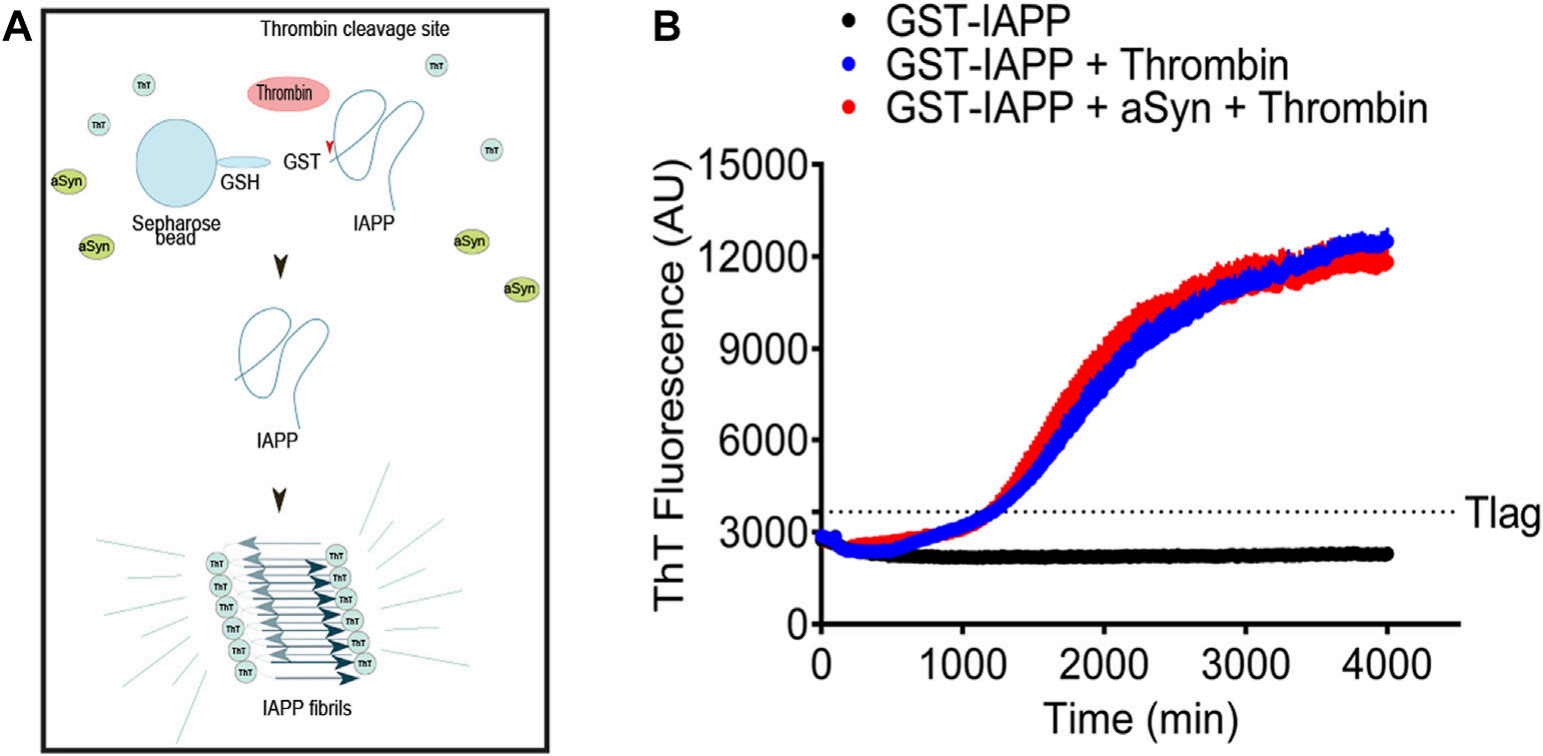
Monomeric aSyn does not affect IAPP fibrillation. **(A)** Schematic drawing of ThT assay on glutathione S-transferase tagged IAPP (GST-IAPP). GST-IAPP with thrombin cleavage site in linker region was expressed and bound to glutathione-Sepharose beads. Bead-GST-IAPP was then incubated with or without aSyn for 60 min at room temperature. The addition of thrombin resulted in the release of monomeric IAPP, which sequentially misfolded and formed amyloid fibrils. **(B)** When bead-GST-IAPP alone or bead-GST-IAPP preincubated with 5 µM aSyn was cleaved by thrombin, the formation of IAPP amyloid fibrils entered elongation phase at similar time points (1220 min and 1200 min) in both samples. Without the addition of thrombin, bead-GST-IAPP did not form detectable amyloid fibrils. The analysis was done in a black 96-well plate, and the ThT signal was measured at 480 nm emission with 440 nm excitation at 20 min intervals. Each data point represented mean ± SD (*n* = 4).

IAPP and aSyn are well-known amyloid-forming proteins. Based on the suggested epidemiological link between T2D and PD (Hu et al., 2007; Schernhammer et al., 2011), it is possible that the proteins can influence each other’s ability to aggregate and form fibrils. IAPP is known to be challenging to solubilize into monomers, and the peptide possesses a high tendency to misfold and form fibrils already at a low concentration. From earlier studies, we know that fusion of IAPP to GST (GST-IAPP) effectively prevents the fibrillation of IAPP (Paulsson et al., 2008). The layout of the experiment is explained in Figure 3A. The expression of IAPP linked to GST (GST-IAPP) prevents fibrillation of IAPP. The linkage between GST and IAPP consists of the thrombin cleavage site LVPR/GS, and the addition of thrombin results in the release of IAPP, which by now can start to fibrillate. In this system starts IAPP fibrillation after a nearly 20 h-long lag phase (Figure 3B blue curve). To allow IAPP and aSyn to interact before fibrillation, the bead-GST-IAPP and 5 µM monomeric aSyn were mixed and incubated at room temperature for 1 h before adding thrombin. Despite the extended incubation time, the addition of monomeric aSyn to bead-GST-IAPP did not change the fibril formation profile of the released IAPP (Figure 3B, red curve). Instead, the kinetics of IAPP fibril formation with or without aSyn were comparable. As expected, in the control group, GST-IAPP without the addition of thrombin did not result in amyloid fibril formation (Figure 3B, black curve).

### 2.4 Preformed aSyn fibrils seed IAPP fibril formation *in vitro*

**FIGURE 4:**
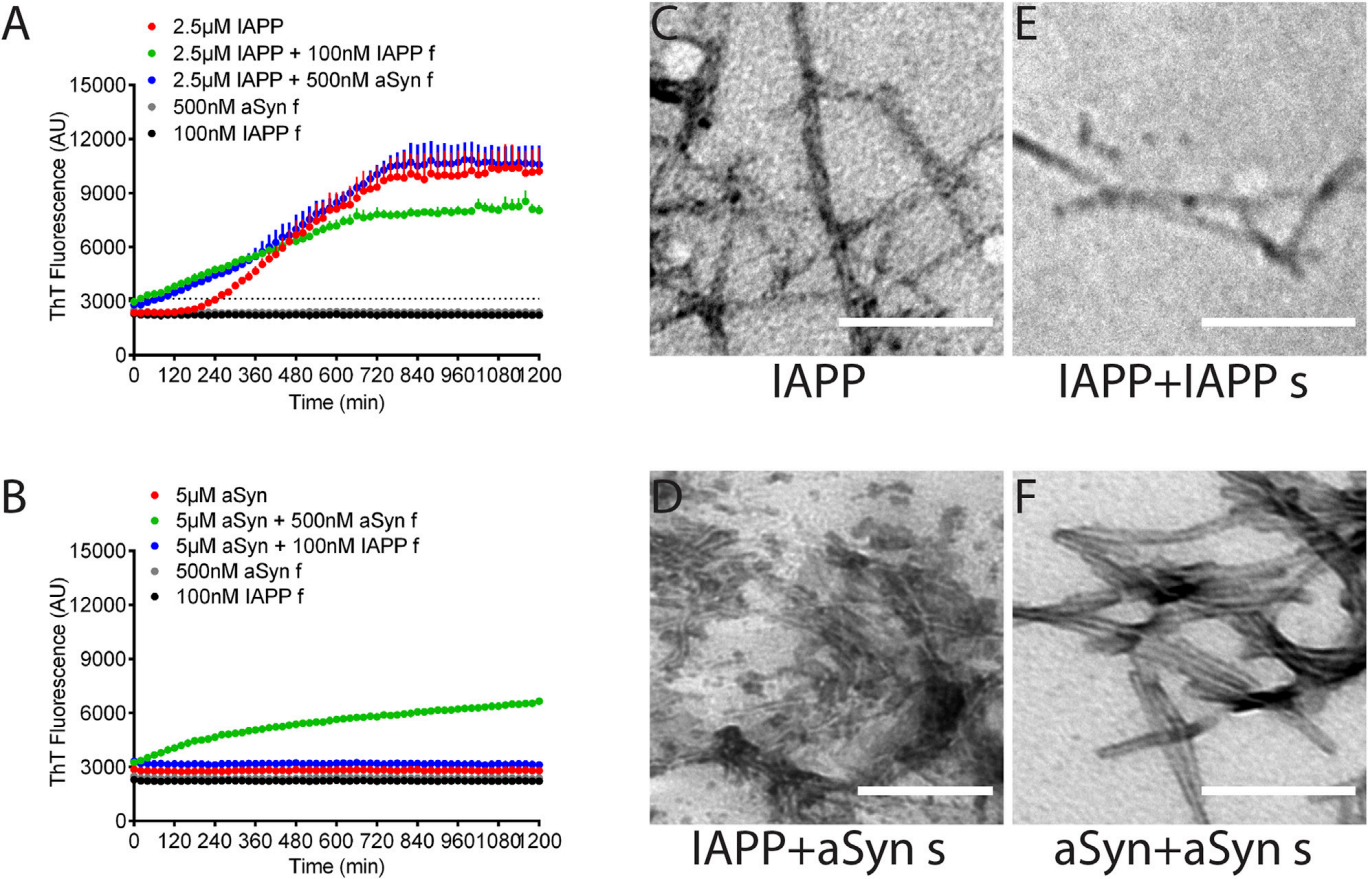
aSyn seeds IAPP fibril formation, not *vice versa*. **(A)**, The lag phase of IAPP fibril formation was 4 h (red). The addition of IAPP seeds drastically reduced the lag phase to 20 min (green), while the addition of aSyn seeds reduced the lag phase to 80 min (blue). **(B)** ASyn alone did not form fibrils under the examined experiment condition (red). Adding aSyn seeds induced aSyn fibril formation (green), while adding IAPP seeds did not induce aSyn fibril formation (blue). Each data point represents mean ± SD (*n* = 4). Electron micrographs visualize amyloid fibrils formed by **(C)** IAPP, **(D)** IAPP with aSyn seeds, **(E)** IAPP with IAPP seeds, and **(F)** aSyn with aSyn seeds. Scale bar: 200 nm.

Seeding and cross-seeding of IAPP and aSyn were performed on synthetic peptides in monomeric form with the addition of preformed fibrils as seeds. At first, different concentrations of sonicated seeds were tested for their efficiency in homologous seeding. Then, based on the results, the concentrations 100 nM IAPP and 500 nM aSyn seeds were selected for further studies. The assay was done in PBS pH 7.4 with 10 μM ThT, and changes in fluorescence were monitored at excitation 440 nm and emission 480 nm. The monomeric IAPP (2.5 µM) had a lag phase of 240 min (Figure 4A, red curve), which, as expected, was dramatically reduced to 20 min by the addition of IAPP seeds corresponding to 100 nM monomeric peptide (Figure 4A, green curve). Also, the addition of aSyn seeds corresponding to 500 nM monomeric peptide shortened the lag phase to 80 min (Figure 4A, blue curve). Fluorescent signals of IAPP seeds or aSyn seeds alone did not change during the 20 h duration of the experiment (Figure 4A, grey and black curves).

Unlike IAPP, monomeric aSyn (5 µM) alone did not form amyloid fibrils under the current experimental condition (Figure 4B, red curve). However, the addition of preformed aSyn seeds (500 nM) to monomeric aSyn (5 µM) triggered fibril formation and almost eliminated the lag phase for aSyn (Figure 4B, green curve). On the contrary, the addition of IAPP seeds corresponding to 100 nM seeds to monomeric aSyn did not trigger aSyn fibril formation (Figure 4B, blue curve). Therefore, under the given experimental condition, preformed seeds of aSyn trigger IAPP fibril formation, although without reaching the same efficacy as obtained for homologous seeding. The addition of preformed IAPP seeds to a monomeric aSyn solution did not cause fibril formation, while homologous seeding was a very efficient trigger for fibril formation. After the ThT-assay was completed, the fibrillar materials were collected, placed on formvar-coated cu-grids, and contrasted with 2% uranyl acetate in 50% ethanol. TEM analysis did not reveal apparent differences between unseeded and seeded fibrils (Figures 4C–F), but IAPP fibrils appeared more twisted than aSyn fibrils.

### 2.5 Knockdown of aSyn with siRNA does not induce toxicity in EndoC-βH1 cells

**FIGURE 5:**
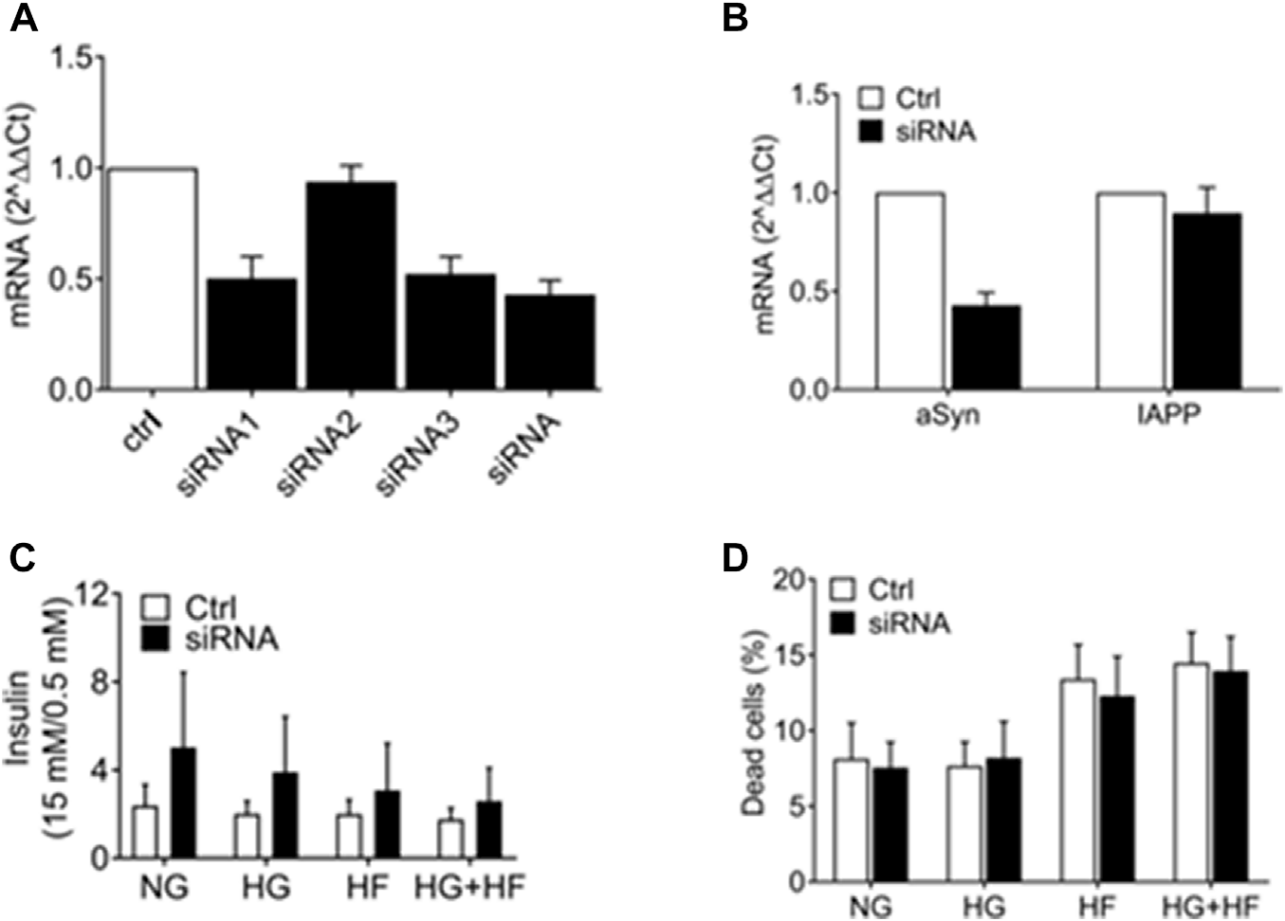
aSyn knockdown did not affect insulin secretion in EndoC-βH1 cells. The efficiency of 3 different aSyn siRNAs was tested in EndoC-βH1 cells, and mRNA levels were analyzed at 48 h post-transfection. **(A)** SiRNA1, siRNA3, and pooled siRNA (siRNA) reduced aSyn mRNA level by 50%. SiRNA2 did not reduce aSyn mRNA level. **(B)** SiRNA reduced aSyn mRNA level while did not alter IAPP mRNA level. Data were normalized to GUSb and correlated to control siRNA. Transfected EndoC-βH1 were exposed to normal glucose (5.5 mM, NG), high glucose (20 mM, HG), high fat (1.5 mM sodium palmitate, HF) or high glucose plus high fat (20 mM glucose and 1.5 mM sodium palmitate, HG + HF) condition for 48 h before being analyzed. **(C)** Glucose-stimulated insulin secretion (GSIS) was analyzed using alphaLISA, where no significant difference in GSIS fold change was observed between the control and siRNA-treated group in all 4 culture conditions (NG, HG, HF, HG + HF). **(D)** The viability of cells was analyzed by propidium iodide (PI) staining using flow cytometry, where no significant difference in dead cell proportion was observed between the control and siRNA-treated group in all 4 (NG, HG, HF, HG + HF) culture conditions. Two-way ANOVA with Bonferroni’s multiple comparison test, mean ± SD, *n* = 3.

We investigated if reducing aSyn has deteriorating effects on the β cells. Three different aSyn siRNAs were tested individually and pooled together for knockdown of aSyn in EndoC-βH1 cells. Scrambled siRNA was used as a control. The aSyn mRNA level was confirmed to be reduced by 50% in EndoC-βH1 cells using siRNA1 (0.502 ± 0.098), siRNA3 (0.521 ± 0.078), and pooled siRNA (0.428 ± 0.064) analyzed 48 h after transfection (Figure 5A). No reduction in IAPP mRNA level was detected in cells transfected with scrambled siRNA (Figure 5B). We next investigated if a decrease in aSyn affected insulin secretion by transfecting EndoC-βH1 cells with pooled siRNA. Six hours after transfection, culture conditions were changed to a medium containing 5.5 mM glucose (NG), 20 mM glucose (HG), 1.5 mM sodium palmitate (HF), or 20 mM glucose + 1.5 mM sodium palmitate (HG + HF) for 48 h. After that, glucose-stimulated insulin secretion (GSIS) was analyzed. Culture in high glucose and increased concentration of free fatty acids is known to stress β cells. The result is presented as fold changes, and although a trend in increased insulin release in cells treated with aSyn siRNA was observed, we failed to determine any statistically significant differences in insulin secretion between the aSyn siRNA-treated group and the control group regardless of which of the four culture conditions was used (Figure 5C). Also, the viability of EndoC-βH1 cells exposed to the same culture conditions was evaluated using propidium iodide (PI) staining and flow cytometry. No differences in dead cell proportion between the siRNA-treated and control groups were observed, independent of culture conditions (Figure 5D). Taken together, a reduction in aSyn in EndoC-βH1 cells had no adverse or advantageous effects on insulin secretion.

### 2.6 Knockdown of aSyn with siRNA does not affect exocytosis rate in EndoC-βH1 cells

**FIGURE 6:**
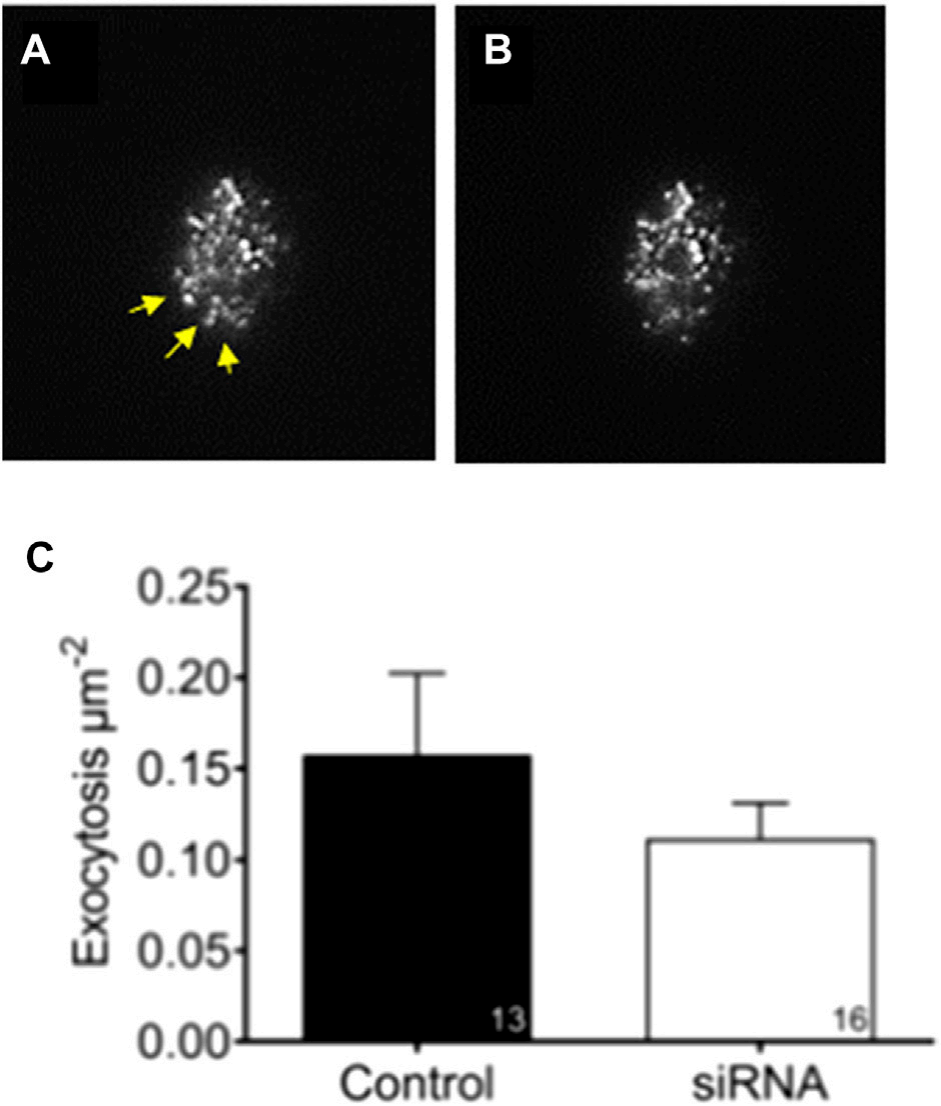
Knockdown of aSyn have no effect on glucose stimulated exocytosis. To study if knockdown of aSyn changes the exocytosis rate at a single granule level, EndoC-βH1 cells were transfected with aSyn siRNA before being infected with adenovirus coding for NPY-tdmOrange2 granule marker. Images were captured prior to and during glucose stimulation using TIRF microscopy. **(A)** An image captured prior to secretion and yellow arrowheads points to the region of interest. **(B)** An image captured during glucose stimulation shows that granules were released **(C)** The exocytosis rate between siRNA treated group (0.158 ± 0.045) and the control group (0.112 ± 0.019) was comparable. Student’s t-test, mean ± SD.

To investigate if reduced levels of aSyn influence exocytosis rate at a single granule level, EndoC-βH1 cells were transfected with aSyn siRNA before being infected with adenovirus coding for the granule marker NPY-tdmOrange2. The exocytosis rate in transfected and transduced cells was monitored with TIRF microscopy (Figure 6A) to 0.158 ± 0.045 in the siRNA-treated group and 0.112 ± 0.019 in the scrambled-treated control group (Figure 6B).

### 2.7 Overexpression of aSyn did not affect viability in EndoC-βH1 cells

**FIGURE 7:**
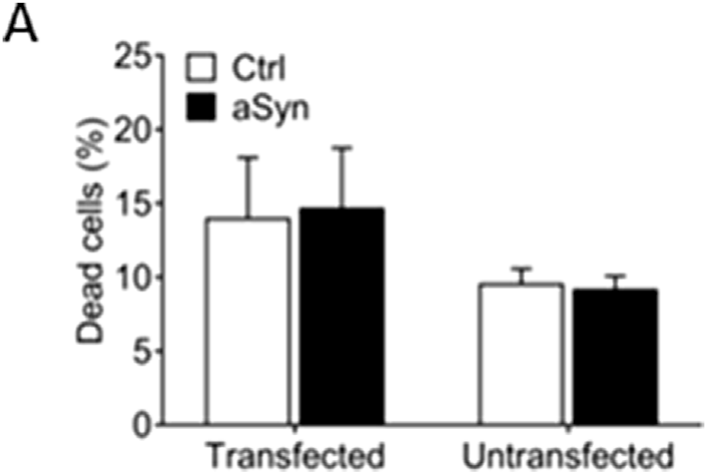
Overexpression of aSyn has no effect on cell viability. **(A)** INS-1 cells were transfected with an aSyn-VENUS vector or control VENUS vector for 48 h before being analyzed. There was no difference in dead cell proportions between the aSyn-VENUS transfected group and the control group. Student’s t-test, mean ± SD., *n* = 3.

We investigated if overexpression of aSyn in β cells had beneficial or adverse effects on cell viability. INS-1 cells were transfected with an aSyn-Venus vector or control Venus vector for 48 h, and cell viability was evaluated using PI staining and flow cytometry. The dead cell proportions in cells transfected with aSyn-Venus and Venus alone were 14.053 ± 4.052 and 14.711 ± 4.045, respectively. Similarly, the cell death proportion in cells transfected with aSyn-Venus and Venus alone were 9.609 ± 0.960 and 9.225 ± 0.858, respectively (Figure 7A).

## 3 Discussion

In the current study, we demonstrate that aSyn exists in β cells, PP cells, and some α cells in human pancreatic islets and in EndoC-βH1 cells. More importantly, we used pancreas tissues from patients with T2D and islet amyloid and showed that aSyn co-localizes with IAPP in human pancreatic islets, but aSyn is absent in extracellular amyloid deposits (Figures 2A, B). These findings are, in part, in line with results from a retrospective autopsy-based study performed on pancreatic tissues from patients diagnosed with incidental Lewy body disease, PD, dementia with Lewy bodies, or with no neuropathological symptoms, and all groups included some patients with T2D. Even though the study group with ¨no neuropathological symptoms¨ included 34 subjects with T2D, IAPP reactivity was described to be extensively present in the β cells, and co-localization of IAPP and aSyn was limited to β cells (Martinez-Valbuena et al., 2018). The absence of IAPP amyloid in pancreas from patients with T2D is difficult to understand.

Structural similarities are believed to be a prerequisite for seeding to occur. The Aβ peptide exhibits areas with significant similarities to human IAPP (O’Nuallain et al., 2004). Kapurniotu et al., (Andreetto et al., 2010), have identified regions in IAPP and Aβ with high interaction capacity, including residues SNKGAII (Han et al., 1995; Iwai et al., 1995; O’Nuallain et al., 2004; Paulsson et al., 2008; Schernhammer et al., 2011; Martinez-Valbuena et al., 2018; Nilsson et al., 2018) of Aβ and NNFGAIL (Sandyk, 1993; Han et al., 1995; Hu et al., 2007; Horvath and Wittung-Stafshede, 2016; Nilsson et al., 2018; Pagano et al., 2018; Mucibabic et al., 2020) of IAPP. Both regions contain the sequences shown to be important for amyloid formation. We used BiFC and confirmed the interaction between IAPP and Aβ, and with the same system, we could identify further propagation of Aβ/IAPP into amyloid (Wang and Westermark, 2021). We used the same technique to investigate molecular interaction between IAPP and aSyn. The ¨key region¨ for aSyn aggregation is identified as aSyn 69–79 or aSyn 71–81 (Giasson et al., 2001; el-Agnaf and Irvine, 2002). The region is present within the aSyn 61–95 fragment selected for the BiFC analysis. Flow cytometry analysis of transfected cells shows that heterologous expression of aSyn (aSVN + aSVC) and IAPP (IVN + IVC) was preferred and resulted in a fluorescent signal in 29.3% and 19.7% of the transfected cells, respectively. At the same time, the number of fluorescent cells decreased to 9.8% and 11% after transfection with aSVN + IVC and IVN + aSVC, respectively. In this BiFC assay, homologous aSyn interaction was more favorable than heterologous aSyn/IAPP interactions. The intensive dot-like fluorescence that developed in cells expressing Aβ/IAPP could not be seen. This is in line with earlier reports on aSyn-aSyn interaction studies using BiFC where no amyloid formation has been detected (Frey et al., 2021). The results argue against the formation of amyloid composed of IAPP and aSyn. Since the isoelectric point for IAPP is above 8 and around 4.67 for aSyn, it is possible that peptide charge is responsible for the interaction.

Earlier, we demonstrated that the expression of IAPP linked to a GST tag prevents the peptide from misfolding and forming amyloid (Paulsson et al., 2008). This can be an advantage in fibrillation studies since the lag phase can be extended. Herein, we have mixed monomeric aSyn with GST-IAPP to facilitate interaction between the two molecules. Upon enzymatic release of IAPP from the GST tag, aggregation was initiated after a 20-h lag phase. The ThT emission profile obtained for aSyn/IAPP was identical to the ThT emission profile observed from samples containing IAPP alone and argued against interaction between the peptides.

The ThT assay (Gade Malmos et al., 2017) is a gold standard technique for studies of amyloid fibril formation, a process known to be condition dependent. The solubilization of the peptide and peptide concentration are both important speed regulators, and aSyn is usually studied at concentrations ranging from 70 to 200 µM (Fagerqvist et al., 2013; Ranjan and Kumar, 2017) and IAPP at concentrations ranging from 2 to 50 µM (Sisnande et al., 2015; Pithadia et al., 2016). IAPP is one of the most amyloidogenic peptides identified, while aSyn, if not seeded, requires agitation to speed up the aggregation process.

Co-aggregation of aSyn and IAPP has been studied earlier, but obtained results are both concordant and discordant. In contrast to us, Horvath and Wittung-Stafshede (2016) reported co-aggregation after mixing monomers of IAPP (5 μM) and aSyn (10 μM) and observed a reduction in lag phase from 3 min to 1.5 min. The result suggests potentiation of IAPP aggregation in the presence of aSyn; however, the initial short lag phase complicates the assessment of the result. In a more recent publication by Mucibabic et al. (2020), fibrillation of monomeric IAPP (2 μM) lacked the lag phase, but after mixing aSyn (7 μM) and IAPP (2 μM) monomers, the fibrillation was delayed to 36 min.

In the ThT analysis performed using synthetic IAPP, we used 2.5 µM and achieved a lag phase of 240 min, supporting the solubilization of the peptide. In the cross-seeding experiments, the addition of preformed aSyn seeds (corresponding to 500 nM) to monomeric IAPP reduced the lag phase to 80 min, and the result suggests that IAPP can be seeded by aggregated aSyn. In an experiment with a similar design, Horvath and Wittung-Stafshede (2016) showed that the fibrillation of 5 μM IAPP was delayed by the addition of preformed aSyn fibrils (corresponding to 1.4 μM peptide), and the lag phase was extended from 3 to 18 min. Despite the use of different concentrations of IAPP and preformed seeds in these two experiments, the ratio between monomers and seeds was comparable, but the results were the contrary. A similar experiment was reported by Mucibabic et al. (2020), and despite the opposite ratio of IAPP (2 μM) and aSyn preformed fibrils (corresponding to 7 μM peptide), they also identified a delayed IAPP fibrillation and the lag phase for IAPP aggregation changed from 0–100 min.

In addition to the observed delayed fibrillation of IAPP in the presence of monomeric aSyn (Mucibabic et al., 2020), the ThT emission profile exhibited a double peak, with the second increase starting after 7 h. The interpretation given by the authors was that aSyn monomers seed IAPP fibrillation. However, because two monomers were mixed, cross-seeding will not occur at the beginning of the analysis. Instead, it is more likely that IAPP fibrils formed early in the analysis trigged fibrillation of aSyn detected as the second peak, and the height of the second peak depended on the concentration of aSyn used. We used 5 uM aSyn and added IAPP seeds (corresponding to 100 nM IAPP) but failed to detect aSyn fibrillation during the 20 h the analysis continued. However, Horvath and Wittung-Stafshede used 70 uM aSyn, and 7 uM preformed IAPP seeds and detected potentiation of the fibrillation with a reduction of the lag phase from approximately 22 h–8 h. The results are inconclusive, but a plausible explanation can be that secondary structures remaining after solubilization affect the fibrillation. Therefore, aiming for a lag phase that extends for more than an hour under unseeded conditions is crucial.

We lysed EndoC-βH1 cells cultured in 5.5 mM glucose and determined IAPP and aSyn concentrations to be 17.6 μg and 28.9 μg per 10^6 cells, respectively. Concentrations used for the ThT assay are well above levels determined in cell lysates, and these experiments are also performed at an inversed relationship. However, while the expression levels of aSyn and phosphorylated aSyn (P-aSyn) were low or below the detection level in patients free of neuropathological symptoms and T2D, subjects with T2D exhibited increased aSyn and P-aSyn immunoreactivity (O’Nuallain et al., 2004). This result implies that the development of T2D causes changes in aSyn expression, and it is not so straightforward to determine IAPP and aSyn ratios.

The biological function of aSyn still needs to be determined. In the brain, aSyn levels regulate the number of dopaminergic neurons (Garcia-Reitboeck et al., 2013), and in the absence of aSyn, a decreased reuptake of dopamine has been reported (Chadchankar et al., 2011). In β cells, aSyn has been implicated in the regulation of basal insulin secretion (Geng et al., 2011), and low levels of circulating aSyn are linked to peripheral insulin resistance (Rodriguez-Araujo et al., 2015).

The subcellular localization for aSyn is described to be cytoplasmatic and associated with the outside of the secretory granules (Acuna et al., 2014; Smaldone et al., 2015). If aSyn is strictly cytoplasmic, aSyn and IAPP will not be in direct contact with each other. We performed double immunolabeling with insulin and aSyn on ultrathin sections, and the TEM analysis revealed secretory granules containing both insulin and aSyn. The presence of aSyn at the same location as IAPP allows for direct interaction between the two molecules, a prerequisite for co-aggregation. However, despite the detection of intra-granular aSyn, no aSyn reactivity appeared in the extracellular amyloid. Previously, in a study on the inhibition of IAPP aggregation, we used PLA and showed that the BRICHOS domain of Bri2 co-localized with IAPP in the secretory granules and also in the extracellular amyloid (Oskarsson et al., 2018). In PLA, a positive signal is generated when the two target proteins are less than 40 nm apart. The distance between tetrameric aSyn present on the outside of the granule and IAPP inside the granule is less than 40 nm apart. Therefore, the obtained PLA signals can derive from antibodies detecting intra-granular aSyn and IAPP, but also aSyn decorating the outside of secretory granules and intra-granular IAPP.

In human islets cultured in 20 mM glucose for 48 h, the IAPP mRNA concentration increased 10-fold, while only a moderate change of 1.18-fold was detected for aSyn. During the development of T2D, hormone secretion is emphasized. We investigated if aSyn has any function in metabolically stressed β cells. SiRNA was used to reduce aSyn in EndoC-βH1 cells. Thereafter, the cells were exposed to metabolic stress (20 mM glucose, 1.5 mM palmitate, or 20 mM glucose and 1.5 mM palmitate) for 48 h. Thereafter, the GSIS was measured, but the reduced concentration of aSyn did not affect insulin secretion. The result contrasts with a previous study, where aSyn knockout increased basal insulin secretion in INS-1 cells (Fagerqvist et al., 2013). This discrepancy could be due to the efficiency of aSyn knockdown; while we had more than 50% reduction of aSyn mRNA, it is still possible that aSyn present in untransfected cells might be sufficient to compensate. However, analysis of the exocytosis rate at a single granule level in EndoC-βH1 cells failed to identify any differences. This experiment allowed for the comparison of exocytosis from EndoC-βH1 cells identified to have reduced aSyn expression and unaffected aSyn expression. The results do not support any adverse effects of aSyn reduction on insulin secretion on cell or granular levels.

Therefore, despite the proximity of aSyn and IAPP in β-cells and the detected capacity of preformed aSyn fibrils to seed IAPP *in vitro*, it is still an open question if an interaction between the two molecules is of pathogenic significance for type 2 diabetes.

## 4 Materials and methods

### 4.1 Cell culture

EndoC-βH1 cells were kindly provided by R. Scharfmann and P. Ravassard (INSERM, Paris, France). EndoC-βH1 cells were cultured in DMEM/F12 medium (Sigma-Aldrich) supplemented with 0.2% (v/v) MycoZap (Lonza, Switzerland), 10 mM nicotinamide (Sigma-Aldrich), 6.7 ng/mL selenite (Sigma-Aldrich), 5.5 μg/mL transferrin (Sigma-Aldrich, MO, United States), 2% (w/v) fatty acid free albumin (Sigma-Aldrich), 50 µM β-mercaptoethanol (Sigma-Aldrich) and 2 mL L-glutamine (ThermoFisher Scientific, MA, United States).

INS-1 and human embryonic kidney 293 (HEK293) (ATCC CRL-1573TM) cells were maintained in RPMI 1640 medium (ThermoFisher Scientific) supplemented with 10% fetal bovine serum (ThermoFisher Scientific), 100 IU/mL penicillin, and 100 μg/mL streptomycin (ThermoFisher Scientific), 2 mM L-glutamine, 1% Sodium pyruvate (ThermoFisher Scientific), and 50 µM β-mercaptoethanol at 37°C with 5% CO2.

### 4.2 BiFC vectors

All BiFC vectors contained three parts: protein of interest, linker, and N-terminal or C-terminal part of fluorescent protein Venus. ASyn (residues 61–95) and human IAPP were amplified using PCR primer pairs containing HindIII or EcoRI restriction sites from existing vectors. A 17-amino acid long linker (RPACKIPNDLKQKVMNH) with restriction sites EcoRI and XbaI was synthesized as the oligonucleotides. N-terminal (residues 1–173) and C-terminal (residues 155–239) parts of Venus were produced with PCR using primer pairs containing XbaI or ApaI restriction sites from existing vectors. After digestion with the corresponding restriction enzymes, all fragments were cloned into a pcDNA3.1 zeo + vector. The insert sequence was verified by sequencing (Eurofins, Luxembourg).

### 4.3 Transfection

HEK293 cells (50,000/well) were seeded in 24-well plates 1 day before transfection. One µg vector was mixed with 2 µl Turbofect transfection reagent according to the user manual (ThermoFisher Scientific). Cells were analyzed 24 h after transfection using an Accuri C6 plus flow cytometer machine (BD Biosciences, NJ, United States).

### 4.4 Human islets culture

Isolated human islets from heart-beating, brain-dead donors were kindly provided by the Nordic Network for Clinical Islet Transplantation (Uppsala University Hospital, Uppsala, Sweden). The regional ethical committee approved the use of human islets in Uppsala, Sweden. Human islets were cultured in CMRL 1066 medium with 5.6 mM glucose supplemented with 10% FCS (Sigma-Aldrich), 100 IU/mL penicillin, and 100 μg/mL streptomycin, 50 µM β-mercaptoethanol and 2 mM L-glutamine at 37°C with 5% CO2.

### 4.5 Production of recombinant IAPP

cDNA corresponding to human iAPP residues 1–37 was inserted behind GST in the expression vector pGEX 4T-1. *E. Coli* Y1090 was used for protein expression initiated with 3 mM Isopropyl β-d-1-thiogalactopyranoside (IPTG) and continued for 3 hours at 25°C with constant shaking at 220 rotations/min. After expression, bacteria were collected and lysed with sonication. Released GST-IAPP was purified using an immobilized glutathione Sepharose column. A detailed instruction is given in reference (Paulsson et al., 2008).

### 4.6 Preparation of aSyn monomer and fibrils

For monomer preparation, recombinant lyophilized aSyn (MJFF, NY, United States) was solubilized in 4.3 mmol/L Na2HPO4, 1.4 mmol/L KH2PO4, 2.7 mmol/L KCl, and 137 mmol/L NaCl (PBS), pH 7.4, to a final concentration of 0.2 mM, aliquoted, and stored at −80°C. For the generation of fibrils, recombinant aSyn (MJFF) was solubilized in PBS to a final concentration of 5 mg/mL (0.35 mM) and agitated on a shaker at 1000 rpm for 1 week at 37°C. The formation of amyloid fibrils was confirmed using Congo red staining. Fibrils were aliquoted and stored at −80°C.

### 4.7 Preparation of IAPP monomer and fibrils

For monomer preparation, synthetic IAPP (Keck Biotechnologies, CT, United States) was solubilized 20 mg/ml in hexafluoro-2-propanol (Sigma-Aldrich), sterile filtered, aliquoted, and evaporated under N2. Aliquoted IAPP was stored at −80°C. For fibril formation, IAPP was solubilized in DMSO (Sigma-Aldrich) and diluted in PBS to a final concentration of 100 μM, and fibrils were allowed to form at room temperature.

### 4.8 ThT assay

Peptide stock solutions of synthetic IAPP (10 µM) and aSyn (20 µM) were prepared in PBS immediately before use. Fibril solutions were sonicated 3 × 10s (Vibra Cell; Sonic & Materials, Danbury, CT) to generate seeds just before the addition to the assay. The assay was performed in 100 µL PBS with 10 μM ThT, and the changes in fluorescence were measured in a FLUOstar Omega microplate reader (BMG Labtech, Stockholm, Sweden) at excitation 440 nm and emission 480 nm at 37°C. The lag phase is presented as t-lag and defined as the point in time where the signal relative to the pre-transition base line has reached 10% of the amplitude of the transition (Arosio et al., 2015).

### 4.9 SiRNA knockdown

EndoC-βH1 cells were seeded in 12-well plate 24 h before transfection. Three different aSyn-siRNAs (siRNA1-5′-GUU AGU GAU UUG CUA UCA U-3′, siRNA2-5′-CUA AHU GAC UAC CAC UUA U-3′ and siRNA3 5′-GGC UUC AAU CUA CGA UGU U-3′ (Sigma-Aldrich) or pooled siRNA1-3 (10 nM of each) were mixed with transfection reagent DharmaFECT (GE Healthcare, IL, United States). Control cells were transfected with 30 nM scrambled siRNA (Sigma-Aldrich). Cells were cultured in a medium containing 5.5 mM glucose (NG), 20 mM glucose (HG), 1.5% mM sodium palmitate (HF) or 20 mM glucose + 1.5% sodium palmitate (HG + HF) for 48 h before being analyzed for GSIS and viability assay.

### 4.10 GSIS

Cells were incubated in 116 mmol/l NaCl, 5.06 mmol/l KCl, 1.007 mmol/l CaCl2, 1.01 mmol/l MgCl2, 1.19 mmol/l KH2PO4, 23.96 mmol/l NaHCO3, 10 mmol/l HEPES, pH 7.4, and 0.2% BSA solution (KRBH) with 0.5 mM glucose for 4 h and rinsed with the same buffer 3 times before being incubated in KRBH with 0.5 mM glucose for 1 h at 37°C with 5% CO2. The medium was collected carefully, and cells were washed with the same buffer 3 times, followed by incubation in KRBH with 15 mM glucose for 1 h at 37°C with 5% CO2. The medium was collected, and GSIS was determined using alphaLISA according to the manufacturer’s protocol (PerkinElmer, MA, United States).

### 4.11 Cell viability test

Cells were collected and rinsed in ice-cold PBS 3 times before incubation in 10 μg/mL propidium iodide (Sigma-Aldrich) and analyzed using flow cytometry (BD Biosciences). The data were analyzed using Flowing software (Turku Centre for Biotechnology, Finland).

### 4.12 Exocytosis assay

EndoC-βH1 cells were seeded on 22 mm uncoated coverslips 1 day before transfection. Cells were transfected with aSyn siRNA or control scrambled siRNA as described above, followed by infection with adenovirus coding for the granule marker NPY-tdmOrange2 (40–45 h). TIRF microscopy on a custom-built Axio Observer Z1 (Zeiss) with a 100×/1.45 oil immersion objective was used to image single granule exocytosis.

### 4.13 Quantitative real-time PCR

Total RNA was isolated from human islets and EndoC-βH1 cells using QIAzol lysis reagent (Qiagen, Germany) and cleaned up with RNeasy MinElute clean-up kit (Qiagen). The concentration was determined with a Nanodrop 2000c (ThermoFisher Scientific), and 1 µg total RNA was used for first-strand cDNA synthesis using oligo (dT) primer according to the manufacturer’s instruction (ThermoFisher Scientific). Primer sequences used for qt-PCR were IAPP (fwd) 5′-TTT​GAG​AAG​CAA​TGG​GCA​TC-3′, IAPP (rev) 5′-CAT​GTG​GCA​GTG​TTG​CAT​TT-3′, α-syn (fwd) 5′-AAT​GTT​GGA​GCA​GTG​GT-3′, α-syn (rev) 5′-TTG​TCA​GGA​TCC​ACA​GGC​AT-3′, GUSb (fwd) 5′-CAC​GGT​GTC​AAC​AAG​CAT​GA-3′ and GUSb (rev) 5′-AAT​CCC​ATA​GCG​GTC​ACA​CA-3'. Each reaction contained 10 ng cDNA, 0.4 µM primer, and 5 µl FastStart Universal SYBR Green Master (ROX) (Roche, Basel, Switzerland), and the analysis was carried out on a QuantStudio 5 Real-Time PCR (ThermoFisher Scientific).

### 4.14 Western blot

Lysates of human islets and EndoC-βH1 cells were mixed with SDS-sample buffer and separated on 16% Tris-Tricine gel. Proteins were transferred to nitrocellulose membranes at 40V, 400 mA for 2 h. The membrane was boiled in 50 mM tris-HCl with 150 mM NaCl, pH 7.4 (TBS) for 5 min, and blocked with 5% milk in TBS for 1 h at room temperature before incubated with primary antibodies Syn-1 (BD Biosciences) and A133 (in-house prepared) diluted in TBS, at 4°C overnight. After rinsing with TBS, the membrane was incubated with HRP-conjugated secondary antibodies diluted in TBS for 2 h. Reactivity was visualized using chemiluminescence (Immobilon, Millipore) and documented with ChemiDoc XRS + system (Bio-Rad, CA, United States).

### 4.15 ELISA

The concentration of aSyn and IAPP in EndoC-βH1 cells was determined using an in-house established ELISA and ELISA EZHA-52 K (Merck Millipore, MA, United States), respectively.

### 4.16 Immunofluorescence

Formalin-fixed paraffin-embedded isolated human islets were incubated with primary antibodies Syn-1 (BD Biosciences, NJ, United States), 1338 (R&D Systems, MN, United States), anti-insulin, anti-glucagon, anti-somatostatin, and anti-pancreatic polypeptide (Agilent Technology, CA, United States), overnight at 4°C. After rinsing with TBS, sections were incubated with secondary antibodies labeled with Alexa Fluor 488 and Alexa Fluor 546 (ThermoFisher Scientific) for 2 h at room temperature.

### 4.17 Proximity ligation assay (PLA)

Formalin-fixed paraffin-embedded human pancreas sections from patients with T2D were incubated with primary antibodies Syn-1 (mouse, diluted 1:500) and IAPP 110 (rabbit, diluted 1:100, Table 1). The reaction was visualized according to the manufacturer’s instruction with Duolink *in situ* reagent (Sigma-Aldrich).

### 4.18 PFTAA-CN staining of islet amyloid

The presence of amyloid was visualized with the LCO pFTAA-CN in sections that underwent immunodetection with PLA. LCO (7.5 μM) is soluble in PBS and does not affect the PLA signal. Sections were incubated in a humidity chamber for 1 h, rinsed in PBS for 3 min × 5 min, and mounted with PBS glycerol.

### 4.19 Immunoelectron microscopy

Islets were fixed in 2% paraformaldehyde with 0.25% glutaraldehyde and embedded in Lowicryl resin (Electron microscopy science, PA, United States). Ultra-thin sections were placed on formvar-coated nickel grids, blocked with 3% albumin (BSA) in TBS for 30 min, and incubated with primary antibodies overnight. The next day, sections were washed for 3 × 5 min and blocked with 3% BSA in TBS for 30 min before incubating with protein-A labeled secondary antibodies for 1 h. Both primary and secondary antibodies were diluted in 1% BSA in TBS. After rinsing with TBS and water, specimens were contrasted by 2% uranyl acetate in 50% ethanol for 10 min and Reynold solution (120 mM sodium citrate, 25 mM lead citrate, pH 12) for 3 min before being viewed using Hitachi H-7100 transmission electron microscope (Hitachi, Tokyo, Japan). Images were captured using Gatan multiscan camera with Gatan digital micrograph software version 3.6.4 (Gatan, CA, United States).

### 4.20 Confocal microscopy

Immunolabelling and BiFC studies were performed using Zeiss LSM780 microscopy (Zeiss, Oberkochen, Germany). PLA and LCO stained sections were analyzed using Nikon Eclipse E800 confocal microscope (Nikon, Kawasaki, Japan). All photos were analyzed using FIJI ImageJ software.

### 4.21 Statistics

All statistical analyses were carried out using GraphPad Prism 8 (GraphPad Software, CA, United States), with *p* < 0.05 considered statistically significant. Student’s *t*-test was used to compare two groups, while one-way ANOVA with Bonferroni’s *post hoc* analysis was used for analysis of experiments with more than two groups.

## Data Availability

The original contributions presented in the study are included in the article/supplementary material, further inquiries can be directed to the corresponding author.
